# Implication of Posttranslational Histone Modifications in Nucleotide Excision Repair

**DOI:** 10.3390/ijms131012461

**Published:** 2012-09-28

**Authors:** Shisheng Li

**Affiliations:** Department of Comparative Biomedical Sciences, School of Veterinary Medicine, Louisiana State University, Baton Rouge, LA 70803, USA; E-Mail: shli@vetmed.lsu.edu; Tel.: +1-225-578-9102; Fax: +1-225-578-9895

**Keywords:** nucleotide excision repair, global genomic repair, histone modifications, acetylation, methylation, phosphorylation, ubiquitination

## Abstract

Histones are highly alkaline proteins that package and order the DNA into chromatin in eukaryotic cells. Nucleotide excision repair (NER) is a conserved multistep reaction that removes a wide range of generally bulky and/or helix-distorting DNA lesions. Although the core biochemical mechanism of NER is relatively well known, how cells detect and repair lesions in diverse chromatin environments is still under intensive research. As with all DNA-related processes, the NER machinery must deal with the presence of organized chromatin and the physical obstacles it presents. A huge catalogue of posttranslational histone modifications has been documented. Although a comprehensive understanding of most of these modifications is still lacking, they are believed to be important regulatory elements for many biological processes, including DNA replication and repair, transcription and cell cycle control. Some of these modifications, including acetylation, methylation, phosphorylation and ubiquitination on the four core histones (H2A, H2B, H3 and H4) or the histone H2A variant H2AX, have been found to be implicated in different stages of the NER process. This review will summarize our recent understanding in this area.

## 1. Introduction

**Figure 1:**
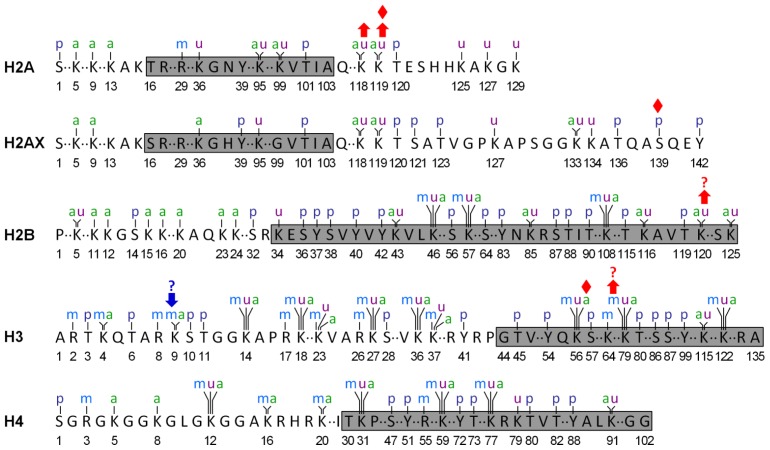
Commonly known posttranslational modifications on the four core histones and the H2A variant H2AX in human cells. Residue numbers below the amino acid sequences are based on the mature (*i.e.*, after posttranslational cleavage of the initiator methione) histones. Residues in the globular domains are shown in the shaded boxes. Modifications shown above the amino acid sequences are: a, acetylation; m, methylation; p, phosphorylation and u, ubiquitination. Data are from PhosphoSitePlus (http://www.phosphosite.org/homeAction.do). Red upwards arrows indicate modifications that have been shown to be required or partially required in the early (prior to incision) stage(s) of nucleotide excision repair (NER); those with a question mark indicate modifications whose roles in NER have not yet directly demonstrated in human cells but are inferred from equivalent modifications in *S. cerevisiae*. Red diamonds indicate modifications that occur at post-incision stage(s) of NER. The blue downwards arrow with a question mark indicates the modification that may suppress NER (inferred from an equivalent modification in *D. melanogaster*). Note that “collective” modifications, especially acetylations and ubiquitinations, at multiple sites on histones may be implicated in NER. The components of these “collective” modifications have not been well-defined and are not indicated in the figure.

Histones are highly alkaline proteins that package and order the DNA into chromatin in eukaryotic cells. The building block of chromatin is the nucleosomal core particle containing a histone octamer (two each of the core histones H2A, H2B, H3 and H4) around which 147 bp of DNA is wrapped [1]. The core histones are predominantly globular except for their unstructured “tails” at the *N*-termini (for H2A also at the *C*-terminus). A huge catalogue of posttranslational histone modifications, including acetylation, methylation, phosphorylation, ubiquitination, sumoylation, ADP ribosylation, arginine deimination and proline isomerization, has been documented [2]. Although a comprehensive understanding of most of these modifications is still lacking, they are believed to be important regulatory elements for many biological processes, including DNA replication and repair, transcription and cell cycle control [2]. They function by influencing chromatin contacts through structural histone changes or influencing electrostatic interactions, or by recruiting non-histone proteins to chromatin. Figure 1 shows the four most common and well known modifications (acetylation, methylation, phosphorylation and ubiquitination) on the four core histones and the H2A variant H2AX.

**Figure 2:**
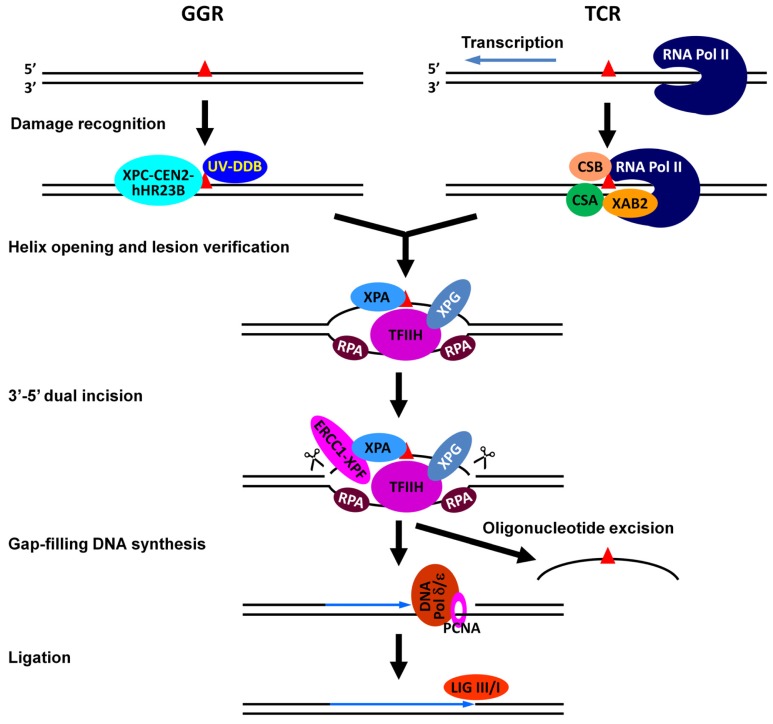
Core factors involved in different stages of NER in mammalian cells.

Nucleotide excision repair (NER) removes a wide range of generally bulky and/or helix-distorting DNA lesions, such as those caused by UV radiation [cyclobutane pyrimidine dimers (CPDs) and 6-4 photoproducts (6-4PPs)], certain genotoxins and cancer chemotherapeutics (e.g., cisplatin) [3]. NER is a multistep reaction that requires the coordinated action of over 30 core proteins implicated in damage recognition, helix opening, lesion verification, dual incision of the damaged strand bracketing the lesion, excision of an oligonucleotide containing the lesion, gap-filling DNA synthesis, and ligation (Figure 2). Global genomic repair (GGR) is an NER subpathway that removes lesions throughout the genome including the nontranscribed strand (NTS) of actively transcribed genes. Transcription coupled repair (TCR) is the other NER subpathway dedicated to rapid repair in the transcribed strand (TS) of actively transcribed genes. The fundamental difference between the two NER subpathways relates to the way that lesions are recognized, the earliest event during the repair process. Lesion recognition during GGR is achieved by specific factors, such as the UV-DDB (UV-damaged DNA-binding protein) heterodimer (containing DDB1 and DDB2/XPE) and the XPC-CEN2-hHR23B complex in mammalian cells [4,5] and Rad7, Rad16, Abf1 and Elc1 in the yeast *Saccharomyces cerevisiae* [6,7]. On the other hand, lesion recognition in TCR is achieved by an RNA polymerase stalled at sites of DNA damage and certain TCR specific factors, such as CSA, CSB and XAB2 in mammalian cells [8,9], and Rad26 in yeast [6]. Following damage recognition, the two NER subpathways use a common set of NER components to complete the repair process.

Although the core biochemical mechanism of NER is relatively well known, how cells detect and repair lesions in diverse chromatin environments is still under intensive research. As with all DNA-related processes, the NER machinery must deal with the presence of organized chromatin and the physical obstacles that it presents [7,10–12]. There have been excellent recent reviews about the roles of certain posttranslational histone modifications in DNA damage response and repair, especially double strand break (DSB) repair [10,11,13–20]. Here, I will summarize our recent findings about the implications of posttranslational histone modifications in NER, especially GGR.

## 2. Histone Acetylation and NER

Seminal studies by Michael Smerdon and colleagues in the 1980’s indicated that histone acetylation might be involved in NER of UV photoproducts. Treatment of cultured human fibroblasts with sodium butyrate, an inhibitor of histone deacetylase (HDAC) that causes hyperacetylation of core histones, results in a marked stimulation of DNA repair synthesis [21]. Also, a “wave” of histone hyperacetylation occurs immediately after UV irradiation and this hyperacetylation phase is followed by a hypoacetylation phase [22]. It was further demonstrated that nucleosomes with a higher level of histone H4 acetylation have a higher level of repair synthesis [23].

Histone acetylation occurs on K residues (Figure 1) and is catalyzed by histone acetyltransferases (HATs) [24]. It appears that HATs are highly diverse and generally contain multiple subunits. The activities and specificities of the catalytic subunit of a HAT depend largely on the context of the other subunits in the complex [24]. Based on their sequence similarities and substrate specificities, nuclear HATs can be grouped into at least three families: GNAT (Gcn5-related *N*-acetyltransferase), p300/CBP (for the two human paralogs p300 and CBP) and MYST (for the founding members *M*OZ, *Y*bf2/Sas3, *S*as2 and *T*ip60) [25]. As will be discussed below, certain members of these HAT families have been shown to be implicated in the early recognition and/or a later stage of NER (Table 1).

### 2.1. Gcn5 and Related Complexes in NER

In the yeast *S. cerevisiae*, Gcn5 is the catalytic subunit of four HAT complexes: ADA, HAT-A2, SAGA and SLIK/SALSA [24]. Gcn5 acetylates K11 and K16 of histone H2B, and K9, K14, K18, K23 and K27 of histone H3 [73–76]. Yeast cells lacking Gcn5 are mildly sensitive to UV and have markedly reduced GGR in certain genes, such as *MFA2* and *MET16* [77,78]. However, these mutants do not have detectable defect in genome-overall NER, indicating that the effect of Gcn5 on GGR is limited to certain locations of the yeast genome [78].

In response to UV-induced DNA damage, K9 and/or K14 of histone H3 are hyperacetylated by Gcn5 in the repressed *MFA2* promoter in yeast [33]. The increased histone acetylation is accompanied by increased accessibility of the DNA template and enhanced GGR [31–33]. The UV-induced K9 and K14 hyperacetylation of histone H3 is independent of Rad4 and Rad14, two factors that are essential for both TCR and GGR [33]. However, the histone H3 hyperacetylation requires the GGR-specific factors Rad7 and Rad16 [31,32]. It appears that both the ATPase and C3HC4 zinc finger (RING finger) domains of Rad16 are required for recruiting Gcn5 to the chromatin in response to UV damage [32]. It was proposed that the GGR complex regulates UV induced histone H3 acetylation by controlling the accessibility of Gcn5 to chromatin. The resultant changes in histone H3 acetylation promote chromatin remodeling necessary for efficient repair of DNA damage [32]. For a recent review as to how histone acetylation by Gcn5 is implicated in NER in yeast, please see the review in this issue [79].

In mammals, GCN5 (also known as KAT2A, GCN5L or hGCN5), the homolog of the yeast Gcn5, also appears to be implicated in NER. GCN5 is a component of the HAT complexes TFTC (TBP-free TAFII complex) and STAGA (SPT3–TAFII31–GCN5L acetylase) [24]. TFTC preferentially binds UV irradiated DNA, free or assembled on nucleosomes and preferentially acetylates histone H3 in nucleosomes assembled on UV damaged DNA [29]. In agreement with this, strong histone H3 acetylation occurs in intact cells after UV irradiation. TFTC is recruited to UV-damaged DNA in parallel with XPA, an essential NER protein involved in damage recognition and verification. TFTC contains SAP130, a pre-mRNA splicing factor that has 50.7% similarity (24.5% identity) and a similar predicted structure to DDB1, a subunit of the UV-DDB complex [29]. SAP130 was shown to have significant binding to UV-irradiated DNA in the absence of other TFTC subunits. However, TFTC binds at least 10 times more efficiently to UV damaged DNA than SAP130 alone, indicating either the damaged DNA binding capability of SAP130 is enhanced in the TFTC complex by additional factors, or another UV-damaged DNA binding activity is present in the TFTC complex [29].

The other GCN5-containing mammalian HAT complex STAGA has also been shown to bind UV damaged DNA [30]. STAGA also contains SAP130. However, in contrast to the report by Brand *et al*. [29], Martinez *et al*. showed that SAP130 alone has no detectable binding activity to UV damaged DNA, and the concentration of SAP130 in STAGA-containing fractions inversely correlates with the STAGA-associated UV damaged DNA binding activity [30]. STAGA interacts with UV-DDB *in vitro* and *in vivo*, and substoichiometric amounts of endogenous UV-DDB components are associated with STAGA, which contrasts with the efficient copurification of SAP130. It was therefore proposed that UV-DDB preferentially interact with a form of STAGA that is not associated with SAP130, and the recruitment of STAGA to UV damaged DNA is accomplished through its interaction with the UV-DDB proteins [30].

Although, both TFTC and STAGA show strong affinity for UV damaged DNA *in vitro*, these complexes may need the assistance of other factors in addition to UV-DDB to gain access to damaged chromatin *in vivo*. In normally cultured human fibroblast cells, while punctuate, GCN5 is distributed throughout the nucleus [27]. Following local sub-nuclear UV irradiation, however, GCN5 is redistributed to discrete areas of the nucleus that overlap with sites of CPDs. The redistribution of GCN5 was shown to be dependent on E2F1, a transcription factor well known for regulation of cell proliferation and apoptosis. In human and mouse cells, both GCN5 and E2F1 are required for rapid acetylation of histone H3 K9 and efficient recruitment of NER factors, such as XPC and XPA, to sites of UV damage to enhance NER efficiency [26–28]. Following UV damage, GCN5 coimmunoprecipitates with E2F1. The ability of E2F1 to promote XPA recruitment and stimulate efficient NER requires residues S31, which can be phosphorylated by ATR or ATM, the dimerization domain, and the marked box domain of E2F1. E2F1 deficiency does not appear to affect the expression of NER factors. However, E2F1 mutants unable to bind DNA or activate transcription retain the ability to stimulate NER, indicating that its function in NER is independent of transcription activation [28].

### 2.2. p300/CBP and Related HATs in NER

p300 and its close homolog CBP (CREB-binding protein), which are present only in multicellular eukaryotes, are often referred to as a single entity, since the two proteins are considered structural and functional homologs [80]. p300/CBP is one of the most potent and versatile acetyltransferases. Unlike other HATs, recombinant p300 and CBP proteins strongly acetylate all four core histones within nucleosomes as well as in free-histone form with little apparent specificity [81].

p300 co-localizes with NER sites shortly after UV irradiation of cultured human fibroblast cells [39]. Microinjection of an anti-p300 antibody to these cells inhibits NER, suggesting that p300 is a HAT involved in the early stage of NER. The recruitment of p300 to NER sites is dependent on p53, “the guardian of the genome”. It appears that, following UV irradiation, p53 recruits p300 (and possibly other HATs) to chromatin, resulting in global histone acetylation and chromatin relaxation, which facilitates lesion detection [39]. The interaction between p53 and p300 is enhanced by ING2, a member of the ING (inhibitor of growth) family proteins [38]. ING2 enhances NER by inducing histone acetylation and chromatin relaxation, which is likely achieved through regulating p53 [42].

p38 MAPK, a family of serine/threonine protein kinases, has been shown to be required for prompt repair of CPDs [82]. The germline stem cell gene *PIWIL2*, which is normally silenced in differentiated cells, is transiently activated after UV irradiation [83]. This activation is associated with NER, because *Piwil2*-deficienct mouse embryonic fibroblasts are defective in NER of CPDs. Like p53, p38 MAPK and PIWIL2 promote histone acetylation and chromatin relaxation. It remains unknown if the roles of p38 MAPK and PIWIL2 in NER are accomplished through regulating p53 and p300.

p300 may also play a role in repair synthesis following the incision step of NER. This function of p300 appears to be achieved through its interaction with PCNA (proliferating cell nuclear antigen), which, by encircling the DNA, acts as a processivity factor for DNA polymerase δ and ɛ in eukaryotic cells [36]. p300 forms a complex with PCNA that does not depend on the S phase of cell cycle, and is associated with freshly synthesized DNA after UV irradiation. By acetylating histones, p300 may participate in chromatin remodeling at DNA lesion sites to facilitate PCNA function in repair synthesis [36]. The HAT activity of p300 and the interaction between p300 and PCNA is regulated by p21, the regulator of cell-cycle progression, transcription and apoptosis [35]. p21 localizes and interacts with both p300 and PCNA at UV-induced DNA damage sites. Loss of p21 or its inability to bind PCNA results in a prolonged binding of p300 to chromatin and an increased association of p300 with PCNA in UV-irradiated cells. Concomitantly, HAT activity of p300 is reduced after DNA damage. The inhibition of p300 HAT activity by PCNA is relieved by p21, which disrupts the association between p300 and PCNA. It was proposed that, after DNA damage, PCNA is first recruited to sites of damaged DNA where p300 is present, thereby inhibiting HAT activity [35]. Immediately afterward, p21 is also recruited, until its local concentration is high enough to bind both PCNA and p300, thereby disrupting their association. The release of p300 restores its HAT activity, thus promoting accessibility of PCNA-dependent repair syntheses in surrounding chromatin regions [35].

The HAT activity of p300 and the interaction between p300 and PCNA is also regulated by ING1b, a splice variant of ING1. Like p21, ING1b also has a PCNA-interacting protein (PIP) domain. UV rapidly induces ING1b to bind PCNA competitively through the PIP domain [40]. In experiments where ING1b was overexpressed and cells were treated with UV to induce interaction of ING1b with PCNA, immunoprecipitates of p300 contained much less PCNA, indicating that high levels of ING1b interfere with association of p300 and PCNA [41]. This is consistent with ING1b having a bridging role between p300 and PCNA because high levels of ING1b would bind independently to both PCNA and protein complexes containing p300, thereby inhibiting the common participation of PCNA, p300 and ING1b within the same protein complex. Furthermore, ING1b has been shown to alter histone acetylation dynamics upon exposure to UV radiation and induces chromatin relaxation to promote NER [37]. Therefore, by regulating p300, ING1b may also play a role in the early stage of NER.

Acetylation of histone H3 K56 is catalyzed by p300/CBP in higher eukaryotes [84] and by Rtt109 (the structural homolog of p300/CBP [85]) in yeast [43–46]. This histone modification has been shown to be stimulated by the histone chaperone ASF1 in all organisms studied. In the yeast *S. cerevisiae*, Rtt109 activity is also stimulated by Vps75, another histone chaperone [46]. Depletion of ASF1 or Rtt109, or abolition of H3 K56 acetylation renders cells sensitive to DNA damaging agents, including UV. However, H3 K56 acetylation does not seem to be required for NER [34]. Instead, the histone modification appears to be important for ordered restoration of chromatin structure and key epigenetic marks following the completion of NER [34]. In response to treatments with DNA damaging agents (UV, ionizing radiation, methyl methanesulfonate, and hydroxylurea), K56-acetylated histone H3 is assembled into chromatin in *Drosophila* and human cells, forming foci that colocalize with sites of DNA repair [84]. It was also observed that a fast initial deacetylation of H3 K56 is followed by full renewal of an acetylated state at ~24–48 h after UV irradiation of human cells [34]. The time course of restoration of H3 K56 acetylation coincides with the removal of UV-induced DNA lesions, making this restoration a potential mark for the completion of NER. ASF1 is crucial for the post-repair restoration of H3 K56 acetylation, which in turn, is needed for dephosphorylation of the H2A variant H2AX and cellular recovery from DNA damage checkpoint arrest [34].

In addition to acetylating histones, p300/CBP also acetylates XPG, the NER factor involved in incision on the 3′ side of a lesion in mammalian cells [86]. Knocking down both p300 and CBP by RNAi or by chemical inhibition with curcumin greatly reduced XPG acetylation, and a concomitant accumulation of the protein at DNA damage sites. The interaction between p300 and XPG is regulated by p21. Human fibroblasts lacking p21 show abnormal accumulation of XPG at DNA damage sites. Therefore, p300/CBP may facilitate NER by acetylating not only histones but also the core NER factor XPG [86].

### 2.3. TIP60 and Related HATs in NER

TIP60 in higher eukaryotes and its homolog Esa1 in yeast belongs to the MYST family of HATs [24,87]. TIP60 specifically acetylates Ks on *N*-terminal tails of histones H2A, H3 and H4, but not H2B. TIP60 and Esa1 have been shown to play a role in repair of DNA DSBs in mammalian [88] and yeast [89] cells, respectively. Although evidence showing the involvement of TIP60 in NER is still lacking, this HAT may at least indirectly play a role in NER in mammalian cells, by modulating histone acetylation and/or the activities of p53 and p21 (two proteins involved in GGR (see above)). TIP60 is sumoylated at K430 and K451 following UV irradiation [47]. This sumoylation promotes the HAT activity of TIP60, leading to enhanced acetylation of histones, such as H2A. Also, p53 and p21 are induced following UV irradiation and this induction is abolished if TIP60 is knocked down or its K430 and K451 are mutated to R to block sumoylation [47].

Esa1 is the only HAT that is essential for yeast cell viability. Viable HAT-deficient *esa1* mutants are sensitive to DNA damaging agents, such as UV, camptothecin, hydroxyuea, methyl methanesulfonate and phleomycin [48]. However, a role for Esa1 in NER in yeast has not been documented. If Esa1 does play a role in NER in yeast, the mechanism may not be the same as that in mammalian cells, as yeast cells lack p53 and p21.

UV irradiation also causes increased histone acetylation in plants, such as corn (*Zea mays*) and Arabidopsis (*Arabidopsis thaliana*) [49,90]. Treatment with curcumin, an inhibitor of histone acetyltransferases, impair repair of CPDs in plants [49]. *A. thaliana* encodes two closely related MYST family proteins, HAM1 and HAM2 (87.9% identity and 92.5% similarity in amino acid sequences), both of which show around 40% identity with human TIP60. Mutations of these HATs, especially HAM1, result in compromised NER [49].

## 3. Histone Methylation and NER

Histone methylation takes place on both K and R residues (Figure 1) [91]. The well-studied methylation residues include K4, K9, K27, K36, and K79 of histone H3, and K20 of histone H4. In general, methylation at K9 and K27 of histone H3 and K20 of histone H4 correlates with transcriptional repression, while methylation at K4, K36 and K79 of histone H3 correlates with gene transcription. Histone methylation is catalyzed by a plethora of histone methyltransferases. Except for the yeast Dot1 and its homologs in higher eukaryotes, all lysine methyltransferases identified so far contain an evolutionarily conserved SET domain [92]. Unlike histone acetylation, which has been known to be implicated in NER for a long time, histone methylation was found to be implicated in NER only in recent years.

### 3.1. Histone H3 K79 Methylation in NER

**Figure 3:**
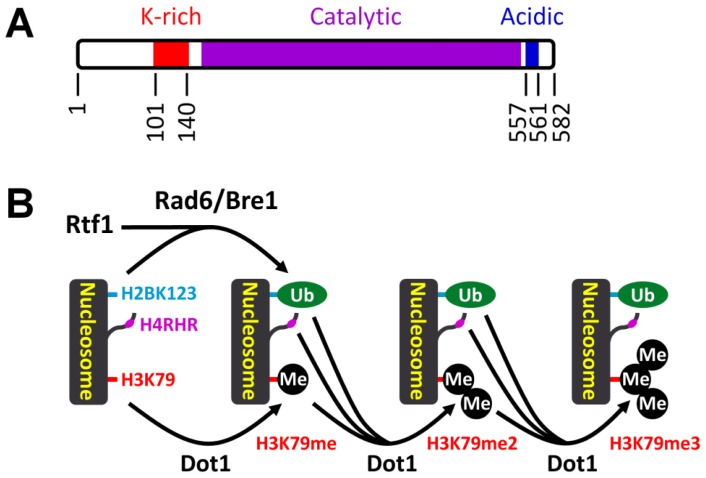
Modulation of Dot1-catalized histone H3 K79 methylation on a yeast nucleosome. A. Domains of the yeast Dot1. B. Modulation of Dot1-catalized histone H3 K79 methylation by trans-activating nucleosomal elements. H4RHR, the short basic patch (residues 17–19, RHR) in the *N*-terminal tail of histone H4. Ub, ubiquitination. Me, methylation. Based on references [100–102,109].

Histone H3 K79 lies within loop 1 of histone H3, which is solvent-accessible and lies adjacent to the interface between the H3/H4 tetramer and H2A/H2B dimer on a nucleosome [1]. In most organisms studied so far, a single Dot1 enzyme seems to be solely responsible for mono-, di- and tri-methylation of histone H3 K79, since knockout of Dot1 in yeast [93] and its homologs in flies [94] and mice [95] results in complete loss of methylation on the site. In contrast, *Trypanosoma brucei,* a parasite that causes sleeping sickness, expresses two Dot1 proteins, TbDOT1A and TbDOT1B, which selectively di- and tri-methylates histone H3 K76 (the equivalent of the yeast and mammalian H3 K79), respectively [96]. Dot1 methylates H3 K79 only on a nucleosome, but not on free histones, indicating that Dot1 needs to crosstalk with certain nucleosomal elements to be active [97,98]. In *S. crevisiae*, two trans-activating nucleosomal elements, mono-ubiquitination of histone H2B K123 [99] and a short basic patch (residues 17–19, RHR) in the N-terminal tail of histone H4 (H4RHR) [100,101], have been found to modulate di- and tri-methylation of H3 K79 (Figure 3). The K-rich domain of Dot1 appears to interact with the mono-ubiquitinated H2B K123 [102], and the acidic domain of Dot1 interacts with the H4RHR [100,101] (Figure 3). Histone H2B K123 mono-methylation is catalyzed by the ubiquitin ligase Bre1 along with the ubiquitin conjugase Rad6 [103,104]. Paf1C, a 5-subunit complex involved in transcription elongation, is required for mono-ubiquitination of H2B K123 by Bre1 and Rad6 [105–107]. It appears that a small region (residues 62–152) of Rtf1, a subunit of Paf1C, can substitute for the complete Paf1C in facilitating global H2B K123 mono-ubiquitination and di- and tri-methylation of H3 K79 in yeast [108].

**Figure 4:**
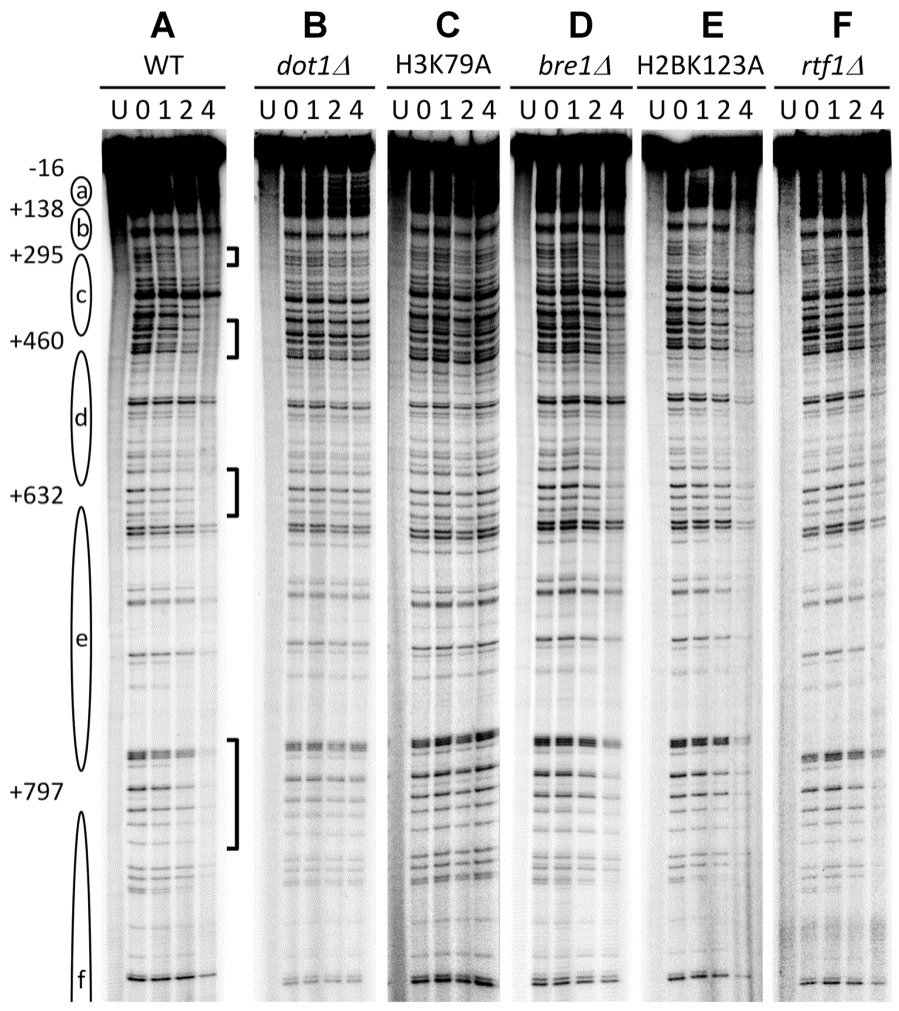
Repair of CPDs in the NTS of the *RPB2* in *S. cerevisiae*. Lanes are DNA samples from unirradiated (U) and 254 nm UV irradiated cells after different times (h) of repair incubation. Ovals on the left represent positioned nucleosomes, with numbers indicating nucleotide positions (relative to the transcription start site) at the centers of the nucleosome linker regions. Brackets on the right of panel A indicate bands of CPDs located in the inter-nucleosomal linker regions that were rapidly repaired in wild type cells. Adapted from [52,63].

Deletion of Dot1 or K79E mutation of histone H3 in yeast causes increased UV sensitivity, indicating that the K79 methylation may play a role in NER or confer tolerance to UV damage [50]. To determine if Dot1 and H3 K79 methylation play roles in NER, we measured repair of UV induced CPDs in yeast by using a nucleotide resolution method [52]. It has been established that NER rates in the nontranscribed strand (NTS) of an active gene reflect GGR [110]. In theory, NER in either strand of an absolutely repressed gene may also reflect GGR. However, low levels of “noise” transcription commonly occur in both strands of supposedly repressed genes in eukaryotic cells [111]. The “noise” transcription cannot be detected by traditional ways, as the transcripts are rapidly degraded by nonsense-mediated decay. However, the “noise” transcription may be able to initiate a certain level of TCR, which can be confused with GGR [112]. Active transcription from the transcribed strand (TS) of a gene may prevent “noise” transcription from the NTS (which is in the opposite direction). Therefore, NER in the NTS of an actively transcribed gene may reflect GGR better than that in either strand of a repressed gene. In wild type cells, CPDs are repaired at different rates at different sites in the NTS of the constitutively transcribed *RPB2* gene (Figure 4A) [52]. The repair rates correlated generally well with nucleosome positioning, being slowest in the central regions of nucleosomal core DNA and fastest in the inter-nucleosomal linker regions. In *dot1Δ* or H3 K79A mutant cells, no obvious repair can be seen (Figure 4B,C), indicating that Dot1 and H3 K79 methylaiton are required for GGR throughout the NTS, including the inter-nucleosomal linker regions. We also found that GGR is decreased in *bre1Δ*, *rtf1Δ* and H2B K123A mutant yeast cells (Figure 4D–F) [52,63], which have decreased di-methylation and undetectable tri-methylation of H3 K79 [52,63]. These results indicate that 1) tri-methylation of H3K79 may contribute to but is not absolutely required for GGR, and 2) lower levels of methylation (mono- and di-methylation) at the K79 also promote GGR. In contrast to their important roles in GGR, Dot1 and H3K79 methylation do not seem to play significant roles in TCR, as *dot1Δ* and H3 K79A mutant cells show no defect in repair of CPDs in the TS of the *RPB2* gene [52].

The underlying mechanism as to how histone H3 K79 methylation mediates GGR remains to be elucidated. In *S. cerevisiae*, ~90% of all histone H3 are methylated on K79 [93,113]. H3 K79 methylation is ~ 10-fold lower (but still 8- to 10-fold higher than background) at all Sir-dependent silenced regions, such as the telomeric and silent mating-type loci, but not at other transcriptionally repressed regions, such as the *TSL1* gene and the promoters of the repressed *SUC2* and *INO1* genes [113]. Histone H3 K79 methylation in the euchromatin may prevent the binding of Sir proteins, thereby limiting gene silencing to heterochromatin (telomeres and silent mating-type loci) [93]. Loss of H3 K79 methylation in the cell leads to promiscuous binding of Sir proteins to the bulk chromatin, resulting in derepression of the normally silent regions. However, loss of H3K79 methylation does not seem to cause promiscuous gene repression, presumably due to limited levels of Sir proteins in the cell. Indeed, loss of H3 K79 methylation has been shown to have no or a very minor effect on genome-wide transcription of genes [114], including NER genes [51], in yeast. It is therefore unlikely that the role of histone H3 K79 methylation in GGR is achieved by modulating expression of NER genes.

Dot1 and H3 K79 methylation have been shown to be required for important aspects of DNA damage checkpoint activation [115]. The roles of Dot1 and H3 K79 methylation in GGR are unlikely to be achieved indirectly by activating the DNA damage checkpoint. First, *dot1Δ* strains largely share the checkpoint defects of *bre1Δ* strains, implying that the checkpoint role of Bre1 (through mono-ubiquitination of H2B K123) is mostly manifested through its ability to permit di- and tri-methylation of H3 K79 [115]. However, although compromised, GGR is still apparent in *bre1Δ* and H2B K123A mutant cells, indicating that di- and tri-methylation of H3K79 contributes to but is not absolutely required for GGR. Second, cells lacking *MEC1*, the homolog of mammalian ATR that plays the most important role in the checkpoint activation in the yeast [116], have little defect in GGR. However, introduction of mutations to *mec1Δ* cells that disrupt H2B K123 ubiquitination or H3 K79 methylation significantly decrease or abolish GGR, respectively, indicating that the histone modifications play much more important roles in GGR than the checkpoint protein [52].

An unmodified H3 K79 covers a small hydrophobic pocket lined by histone H3 L82 and histone H4 V70 [117]. However, a di-methylated H3 K79 assumes an alternative conformation, partially uncovering the small hydrophobic pocket [117]. A mono- or tri-methylated H3 K79 may also assume such an alternative conformation, although crystal structures of nucleosomes containing mono- or tri-methylated H3 K79 have not been available. However, H3 K79 methylation does not seem to affect the local secondary structure of histones, the nucleosome structure or the condensation of nucleosome arrays [117]. Furthermore, cells with the K79E mutation of histone H3 show normal nucleosome positioning and stability in minichromosomes in yeast [118]. It has been proposed that H3 K79 methylation exerts its function through affecting interactions of certain factors with the nucleosome [117]. It is therefore unlikely that the role of histone H3 K79 methylation in GGR is achieved by disrupting the chromatin structure. Rather, the methylated H3 K79 may serve as a docking site for the GGR machinery on the chromatin. However, direct evidence for the interaction between methylated H3 K79 and the GGR machinery remains to be established.

A study showed that yeast cells with the K79R mutation of histone H3 have impaired NER at the transcriptionally silent mating-type locus *HML*, while maintaining nearly normal NER in the constitutively expressed *RPB2* gene and transcriptionally repressed *GAL10* gene [51]. This study collectively measured NER in both strands of the different loci (*i.e.*, did not distinguish the two strands), which may have missed the detection of repair defect in the NTS of the *RPB2* gene. There is evidence that GGR and TCR compete for common NER factors; specific elimination of GGR may enhance the rate of TCR (*i.e.*, repair in the TS) in the cell [112]. The observation that H3 K79 methylation does not affect overall NER in the repressed *GAL10* gene may be due to TCR mediated by “noise” transcription. Indeed, we observed that *rad16Δ* cells, which are completely deficient in GGR, do not show a significant slowdown of overall NER in the repressed genes, such as *GAL1-10*, *ADH2* and *PHO5* [112]. The Rad16-independent NER in the apparently repressed genes, is likely to be due to enhanced TCR mediated by “noise” transcription, as the repair is absolutely dependent on Rad26, the TCR-specific factor [112]. Another report showed that yeast cells with the K79E mutation of histone H3 have normal NER in minichromosomes and slightly enhanced NER in a subtelomeric region [118]. The reason why no significant NER deficiency was seen in the K79E mutant system is unknown. One possibility is that TCR mediated by “noise” and/or aberrant transcription might have contributed to the observed NER in the minichromosomes and subtelomeric region in the K79E mutant cells. Alternatively, the K79E mutation may obviate the requirement of H3 K79 methylation for GGR.

### 3.2. Implication of Other Histone Methylations in NER

In response to UV irradiation, a global decrease in tri-methylation of histone H3 K9 was observed in salivary gland cells in wild type flies (*Drosophila melanogaster*) [53]. Flies with mutations in the Dmp53, the homolog of mammalian p53, have reduced basal levels of H3 K9 tri-methylation but increased levels of H3 K9 tri-methylation after UV irradiation. It appears that UV irradiation enhances the levels of H3 K9 demethylase (dKDM4B) transcript and protein in wild type but not Dmp53 mutant flies. Dmp53 promotes *dKdm4B* transcription by binding to an element of the *dKdm4B* gene in response to UV irradiation. Heterozygous mutants for the *dKdm4B* gene are UV sensitive and deficient in repair of CPDs. Therefore, tri-methylation of H3 K9, which has well-established roles in gene silencing and heterochromatin formation, may inhibit NER. Dmp53 may relieve the inhibitory effect by promoting dKdm4B expression and H3 K9 demethylation [53]. Up to date, the role of histone H3 K9 demethylation in NER and how p53 is involved in the regulation have not yet been documented in other organisms.

In the fission yeast *Schizosaccharomyces pombe*, methylation of histone H4 K20 is catalyzed by Set9 [54]. Methylation of histone H4 K20 appears to play a role in DNA damage response. Loss of Set9 activity or the K20R mutation of H4 markedly impairs cell survival after genotoxic challenge, including UV radiation [54]. In mammalian cells, several enzymes target histone H4 K20 methylation, consistent with distinct mono-, di-, and tri-methylation states controlling different biological outputs. Mouse cells lacking the Suv4-20h histone methyltransferase have only mono-methylated but essentially no di- and tri-methylated H4 K20. The mutant mouse cells are sensitive to DNA damaging agents, including UV, and are defective in repair of DSBs [55]. However, if methylation of histone H4 K20 also plays a role in NER is as yet unknown.

## 4. Histone Phosphorylation and NER

Histone phosphorylation takes place on S, T and Y residues (Figure 1). Upon induction of DNA DSBs by ionizing radiation, histone H2AX, a variant of histone H2A present in higher eukaryotes, is rapidly phosphorylated on S139 by ATM (ataxia-telangiectasia mutated) and DNA-PKcs [119]. Phosphorylated H2AX (γH2AX) marks the site of damage and provides a nucleation site for the formation of DNA damage checkpoint and repair complexes. UV irradiation, which does not directly induce DSBs, also rapidly induces γH2AX in human cells [57,59]. However, different from ionizing radiation, which induces distinct γH2AX foci, UV irradiation mainly induces γH2AX as a diffuse, even pan-nuclear staining. The UV induced γH2AX is primarily mediated by ATR (ataxia-telangiectasia mutated and Rad3-related), rather than ATM [56,58,59]. ATM is reported to be activated by DSBs, whereas ATR is recruited to single-stranded regions of DNA [120].

γH2AX does not seem to significantly affect NER, as S139A mutation in mouse cells has no influence on UV sensitivity [121]. H2A S129 in *S. cerevisiae* is equivalent to H2AX S139 in mammalian cells. H2A S129A mutant yeast cells are only slightly UV sensitive [62]. H2A S129E mutation, which mimicks H2A phosphorylation, does not affect DNA accessibility and NER of UV induced DNA lesions in yeast [122]. However, NER seems to play an important role in the induction of γH2AX by UV. Blockage of DNA repair synthesis by DNA polymerase inhibitors (aphidicolin, HU or cytosine arabinoside) greatly increase the induction of γH2AX by UV, indicating that perturbation of a gap-filling step of NER induces γH2AX, possibly by generating NER intermediates of single stranded DNA gaps [56,58]. Indeed, XP-A and XP-G cells, which are deficient for both TCR and GGR, do not show induction of γH2AX by UV [56–58]. XP-C cells, which are deficient in GGR but proficient in TCR, have no or very little induction of γH2AX. In contrast, the induction of γH2AX by UV is not significantly compromised in CS-B cells, which are specifically deficient in TCR, indicating that GGR contributes much more prominently to γH2AX induction than does TCR [56].

Although NER, especially GGR, is required for rapid and strong induction of γH2AX by UV, NER-deficient cells display certain levels of delayed but persistent γH2AX [72,123,124]. The NER-independent induction of γH2AX coincides with the formation of single strand DNA breaks by the action of the endonuclease APE1 [124]. γH2AX is the signal for activation of DNA damage checkpoint [125]. The persistence of γH2AX in UV irradiated NER-deficient cells may reflect the persistence of DNA damage (incompletion of NER) [123,124].

Phosphorylation of S10 and T11 of histone H3, which are catalyzed by multiple protein kinases, enhances GCN5-mediated H4 acetylation to promote transcription [126,127]. In mammalian cells, S10 and T11 of hisotne H3 are dephosphorylated following UV irradiation and rephosphorylated after repair of the UV damage [60,61]. In *S. cerevisiae*, S122 and T126 of histone H2A are also dephosphorylated following UV irradiation [62]. If and/or how phosphorylations at these sites are implicated in NER remain to be determined.

## 5. Histone Ubiquitination and NER

Ubiquitin, a 76-amino-acid peptide expressed in all eukaryotic cells, can be covalently conjugated to proteins [128]. Protein ubiquitination can either act as a signal for the 26S proteasome-mediated degradation or modulate molecular characteristics and hence function and/or localization of the substrate protein. Ubiquitination usually occurs via a three-step enzymatic reaction, involving ubiquitin activating (E1), conjugating (E2) and ligating (E3) enzymes that conjugate either one (mono-ubiquitination) or multiple (poly-ubiquitination) ubiquitin moieties on a protein, depending on the substrate-specific combination of used E2 and E3. All four core histones are targets for ubiquitination. However, like those of other types of histone modifications, the precise functions of most histone ubiquitination activities remain obscure.

In *S. cerevisiae*, mono-ubiquitination of histone H2B K123 by the Rad6/Bre1 E2/E3 complex is partially required for GGR, as deletion of the BRE1 gene or the K123A mutation causes compromised repair of CPDs in the nontranscribed strand of an active gene (Figure 4D,E) [52,63]. Ubiquitination of histone H2B K123 may act as a “wedge” to non-specifically open-up the chromatin and allow access to other factors, such as Set1-COMPASS and Dot1, two histone methyltransferase responsible for methylation of histone H3 K4 and K79, respectively [129]. Alternatively, the ubiquitination may serve as a bridge for recruitment of other proteins. The latter possibility is supported by the observation that H2B mono-ubiquitination actually stabilizes the chromatin structure by preventing the constant eviction of H2A-H2B from the chromatin [130,131]. The mechanism of how ubiquitination of H2B K123 facilitates GGR remains to be elucidated. One possibility is that the ubiquitination plays a role in GGR indirectly, by enabling di- and tri-methylation of H3 K79 (see above, section 3.1) [52,63]. In mammalian cells, the role of mono-ubiquitination of histone H2B K120 (the equivalent of yeast H2B K123) in NER has not yet been documented.

The UV-DDB heterodimer (containing DDB1 and DDB2/XPE) is part of a larger cullin-RING ubiquitin ligase complex in higher eukaryotes [132,133]. The ubiquitin ligase activity of the complex is transiently activated by UV irradiation and is specifically directed to chromatin at damaged sites [69]. Several proteins are ubiquitinated by the ubiquitin ligase complex upon UV exposure, including the core histones H2A [64,65], H2B [66], H3 and H4 [66,67], XPC [68] and DDB2/XPE itself [69]. The ubiquitination sites on H2A are K118 and K119 (also numbered as K119 and K120 if the posttranslationally cleaved initiator methione residue is counted as residue 1) [65,134]. However, the exact sites of ubiquitination on other core histones have not yet been identified. The ubiquitination of histones H2A [134], H3 and H4 [67] by the ubiquitin ligase complex destabilizes nucleosomes, which facilitates release of polyubiquitinated DDB2/XPE and may enable NER factors to get access to the lesions. Additionally, the ubiquitination of histones could constitute a signal for the assembly of NER factors. Indeed, the NER protein hHR23B, which forms the damage recognition complex with XPC, has two ubiquitin-associated domains that bind ubiquitin [135,136].

While histone H2A is transiently ubiquitinated by the UV-DDB-containing ubiquitin ligase complex to initiate NER, this core histone is also strongly ubiquitinated on K119 by a different ubiquitin ligase after the incision step of NER in human cells [70–72]. Although the post-incision ubiquitination of H2A coincides with induction of γH2AX, γH2AX is not required for H2A ubiquitylation. It was initially observed that the post-incision ubiquitination of H2A is dependent on the Ring2 (also known as Ring1B) ubiquitin ligase, as the ubiquitination was strongly reduced in cells where the Ring2 was knocked down [70]. However, the Ring2 ubiquitin ligase does not localize at sites of UV DNA damage [72]. It was later found that the RNF8 (E3) ubiquitin ligase and the Ubc13 (E2) ubiquitin conjugase are responsible for the post-incision ubiquitination of H2A [71]. Depletion of RNF8 and Ubc13 causes UV hypersensitivity without affecting the rate of NER, indicating that these factors and the post-incision ubiquitination of histone H2A function downstream of the NER process. RNF8 is recruited to the sites of UV damage in a MDC1-dependent manner, similar to its recruitment to DSBs, but requires ATR (instead of ATM) as well as single stranded NER intermediates. Thus, there may be a conserved common pathway of H2A ubiquitination for both UV lesions and DSBs. The H3–H4 chaperone CAF-1, which mediates DNA synthesis-dependent nucleosome assembly, has also been shown to be required for formation of ubiquitinated H2A foci at locally UV irradiated sites [20]. Therefore, the H2A ubiquitination may occur at newly assembled nucleosomes following DNA repair synthesis, or the ubiquitinated H2A is deposited on the repaired regions from an existing pool [20]. RNF8 is required for recruitment of 53BP1 and BRCA1 to the sites of UV damage and for activation of the checkpoint protein Chk1 [71]. Histone H2A appears to be continuously ubiquitinated by RNF8 as long as DNA lesions, including the poorly repaired CPDs, are present. Through activating and maintaining the DNA damage checkpoint, the sustained ubiquitination of H2A by RNF8 may prevent the progression of a cell cycle before the completion of DNA repair [71].

## 6. Concluding Remarks

Ordered and well-coordinated posttranslational histone modifications may be implicated in the whole process of NER. In view of the diversity and enormous possible combinations of the posttranslational histone modifications, the ones that are currently known to be implicated in NER may only represent a tip of an iceberg and the “code” of histone modifications for NER is far from being clear. More histone modifications and relevant modifying and modulating enzymes involved in the repair process may remain to be identified and characterized. How the different histone modifications crosstalk and coordinate with each other, with histone remodeling complexes and with NER factors at different stages of the repair process remains to be fully understood. It is also very important to distinguish which modifications are truly involved in NER and which are just “passenger” modifications that gratuitously occur during the NER or NER-related DNA damage response. Furthermore, NER in different regions/domains of the genome may require different histone modifications (or combinations of histone modifications). For example, the HAT Gcn5 appears to be required for GGR in certain locations of the genome but not for overall GGR in yeast [78]. For multicellular organisms, the radial and spatial distributions of individual chromosomes in the nucleus [137] and histone modifications in different regions/domains of each chromosome can be significantly different among different cell types [138]. It is therefore reasonable to believe that different cell types may have different requirement of histone modifications at different region/domain of the genome. This illustrates the need for high-resolution genome-wide assessment of DNA damage, repair and histone modifications [79,139]. Only then will we be able to paint a complete picture regarding the implication of posttranslational histone modifications in NER.

## Figures and Tables

**Table 1:** Histone modification enzymes and resultant histone modifications implicated in NER.

Modification enzymes	Modulators	Histone modifications	Organisms	Stage(s) of NER implicated	Ref.
**Histone acetylation**					
GCN5	UV-DDB, E2F1	H3, H4	*H. sapiens, M. musculus*	After damage recognition by UVDDB	[26–30]
Gcn5	Rad7, Rad16	H2B, H3, H4	*S. cerevisiae*	After damage recognition by Rad7/Rad16	[31–33]
p300/CBP	p53, p21, ING1b, ING2, ASF1	H2A, H2B, H3, H4	*H. sapiens, M. musculus*	Chromatin relaxation prior to and after lesion detection; repair synthesis; chromatin restoration after NER	[34–42]
Rtt109	Asf1, Vps75	H3 (K9, K56)	*S. cerevisiae*	?	[43–46]
TIP60		H2A, H4	*H. sapiens*	Chromatin relaxation prior to and after lesion detection?	[47]
Esa1		H2A, H4	*S. cerevisiae*	?	[48]
HAM1			*A. thaliana*	A stage before incision	[49]
HAM2			*A. thaliana*	A stage before incision	[49]
**Histone methylation**					
Dot1	Bre1, Rad6, Rtf1	H3 (K79)	*S. cerevisiae*	A stage before incision	[50–52]
dKDM4B	Dmp53	H3 (K9) demethylation	*D. melanogaster*	A stage before incision	[53]
Set9		H4 (K20)	*S. pombe*	?	[54]
Suv4-20h		H4 (K20)	*M. musculus*	?	[55]
**Histone phosphorylation**					
ATR		H2AX (S139)	*H. sapiens, M. musculus*	After incision; chromatin restoration after NER	[56–59]
Multiple kinases (e.g., TPL-2 and DAP kinase 3)		H3 (S10, T11)	*H. sapiens, M. musculus*	?	[60,61]
?		H2A (S122, T126)	*S. cerevisiae*	?	[62]
**Histone ubiquitination**					
Bre1	Rad6, Rtf1	H3 (K123)	*S. cerevisiae*	A stage before incision	[52,63]
UV-DDBcullin- RING ubiquitin ligase		H2A (K118, 119), H2B, H3, H4	*H. sapiens, M. musculus*	Chromatin destabilization and possible recruitment of lesion recognition factors after initial lesion detection by UV-DDB	[64–69]
RNF8	UBC13, CAF-1	H2A (K119)	*H. sapiens*	During or after repair synthesis; chromatin restoration after NER	[70–72]
